# AI-human collaborative approaches in emotional training: applying visual thinking strategies as an analytical framework for medical students’ drawings from art-based activities

**DOI:** 10.1186/s12909-025-08165-9

**Published:** 2025-12-03

**Authors:** Hui-Ching Weng, Chung-Ying Lin, Xi-Zhen Ke, Yu-Ruei Kuo

**Affiliations:** 1 Institute of Allied Health Sciences, College of Medicine, National Cheng Kung University, 1 University Road, East Dist, Tainan City, 701401 Taiwan; 2 Institute of Gerontology, College of Medicine, National Cheng Kung University, Tainan, Taiwan; 3 Biostatistics Consulting Center, College of Medicine, National Cheng Kung University Hospital, National Cheng Kung University, Tainan, Taiwan; 4 School of Nursing, College of Nursing, Kaohsiung Medical University, Kaohsiung, Taiwan; 5 Department of Accountancy, College of Management, National Cheng Kung University, Tainan, Taiwan; 6 Tri-Service General Hospital and National Defense Medical Center, Taipei, Taiwan; 7 National Defense Medical Center, Taipei, Taiwan

**Keywords:** AI-human collaborative approaches, Emotional training, Empathy, Visual thinking strategies, Metacognitive reflection, Art-based activities, Medical students

## Abstract

**Purpose:**

This study examines AI-human collaborative emotion training for medical students using an art-based intervention with visual thinking strategies (VTS) and metacognitive reflection. It aims to measure pre-post changes in emotion appraisal, empathy, and well-being, along with post-intervention reflective writings.

**Methods:**

A quasi-experimental design compared medical students from an elective course in 2023 (control, *n* = 35) and 2024 (intervention, *n* = 33), for a total of 68 participants. The six-week intervention consisted of art-based activities three hours per week. AI-human collaborative emotion training used VTS as an interpretive framework where participants uploaded digital artwork to AI platforms, corrected AI interpretations, and compared these with peer feedback and their original intent, fostering metacognitive engagement in reflecting on the recognition and perception of both one’s own and others’ emotions. Outcome measures were emotion appraisals recorded using survey and reflective writings.

**Results:**

Self-emotion appraisal significantly improved in the intervention group (pre: 5.13; post: 5.40; *p* = 0.035) but not in the control group (*p* = 0.501). The significant finding remained significant after controlling for age and gender (*p* = 0.027). Others’ emotion appraisal showed no significant changes. Qualitative analysis revealed AI excelled in precise visual details and symbolism, while peers provided holistic, experience-based interpretations.

**Conclusions:**

AI-assisted VTS art analysis, combined with metacognitive reflection, enhanced emotional appraisal, showing how technology and human interaction complement emotional learning, offering a balanced framework for innovation and human engagement in medical education.

**Supplementary Information:**

The online version contains supplementary material available at 10.1186/s12909-025-08165-9.

## Introduction

Medical education faces a critical challenge in balancing professional training with students’ emotional well-being [1]. While medical students are rigorously trained to empathize with patients, their own emotional self-care is often overlooked. This imbalance is reflected in concerning statistics, with burnout rates ranging from 7% to 75.2% and high prevalence of depression (27.2%) and anxiety (39.4%) among medical students [2–4]. Emotional intelligence (EI) has been recognized as a core competency by the Accreditation Council for Graduate Medical Education (ACGME) due to its significant role in fostering empathy, teamwork, communication skills, stress management, and critical thinking in medical practice [5, 6].

A key barrier in medical education is the awareness-implementation gap in emotional training. Despite broad acknowledgment of EI’s role in reducing burnout, it remains underemphasized in curricula. Research indicates that while emotions are fundamental to medical education, their explicit integration into learning design is limited, with only 56% of remediation interventions addressing emotions and just 25% intentionally incorporating them into program structures [7, 8].

Additionally, a digital native-immigrant learning gap complicates modern medical education. Digital natives—students who have grown up with technology—favor visual, interactive learning, whereas digital immigrants—many of their instructors—rely on traditional, text-based methods [9]. Digital natives, skilled in multitasking and rapid adaptation to technological advancements, are reshaping learning approaches in medical education [10]. This shift is further amplified by AI tools like ChatGPT, which offer personalized, efficient learning experiences but struggle to process affective meaning, raising concerns about the authenticity of emotional development in medical training [11, 12].

A technology-emotion integration gap further underscores the complexities of incorporating AI into emotional learning. While AI has demonstrated significant benefits in medical education—85% of studies highlight its advantages in areas such as scientific writing and research efficiency—its role in fostering EI remains uncertain [13]. AI’s increasing involvement in reflective tasks may reduce students’ opportunities for emotional self-discovery and development [12]. This paradox highlights a fundamental challenge: while AI enhances cognitive learning, it may simultaneously impede the cultivation of authentic emotional engagement, revealing a gap between technological advancements and the humanistic aspects of medical education.

To address these gaps, visual thinking strategies (VTS), implemented through art-based activities, has emerged as a promising intervention. VTS employs structured questioning to develop visual literacy, critical thinking, and communication skills [14]. Based on Housen’s model of aesthetic development, VTS guides learners through five interpretative stages: accountive, constructive, classifying, interpretative, and re-creative viewing. The approach is anchored in three core questions: “What’s going on in the image?”, “What do you see that makes you say that?”, and “What more can we find?” These strategies enhance observational and interpretative skills, fostering deeper emotional awareness and empathy. Empirical evidence supports the effectiveness of VTS in medical education.

A systematic review by Cerqueira et al. (2023) found that all three studies assessing empathy in medical students using VTS reported significant improvements [15]. Other studies highlight enhanced emotional awareness and patient understanding through participant feedback. The Art of Empathy curriculum significantly improved medical interns’ compassionate care scores (*p* = 0.039) and enhanced their ability to interpret patients’ emotions and perspectives [16]. A systematic review by Alkhaifi et al. (2023) further corroborates these findings, showing that nearly half (48%) of visual art-based programs improved emotional recognition and perspective-taking, while 57% promoted wellness through stress-relieving art activities [17]. Despite promising qualitative results, many studies relied on subjective assessments rather than validated measures, underscoring the need for more rigorous evaluation methods. Researchers advocate for evidence-based integration of arts in medical training, highlighting its role in developing comprehensive skills like team building, communication, and resilience [18, 19]. Participant-created drawings specifically offer value in uncovering metaphorical insights into emotions and enhancing conversational interviews [20–22]. Higher-level thinking in medical education encompasses disciplined, systematic thinking that goes beyond routine clinical reasoning [23, 24]. As a meta-skill in medical practice, it combines critical and creative thinking alongside clinical intuition, crucial for expert decision-making [25].

Metacognition is the awareness and regulation of one’s cognitive and emotional processes and is closely linked to emotional intelligence, particularly in self- and other-emotion appraisal [26]. By monitoring and evaluating emotional reasoning, learners can refine how they recognize their own emotions and interpret others’ cues. Technological tools such as real-time engagement gauges [27] extend this reflection into dynamic learning settings. Embedding metacognitive elements into art-based VTS activities may therefore strengthen emotion appraisal and support sustained emotional competency.

The present study had two primary objectives. First, it examined the effectiveness of AI-human collaborative emotion training for medical students through art-based VTS, evaluating changes in emotion appraisal, empathy, and well-being. Second, it analyzed students’ reflective writings, comparing AI-generated interpretations of their artwork with peer feedback and their authentic emotional intentions. By exploring the intersection of AI, art-based learning, and emotional development, this study aims to provide insights into how technology and human interaction can be integrated to enhance emotional training in medical education.

## Methods

### Research design and participants

**Fig. 1:**
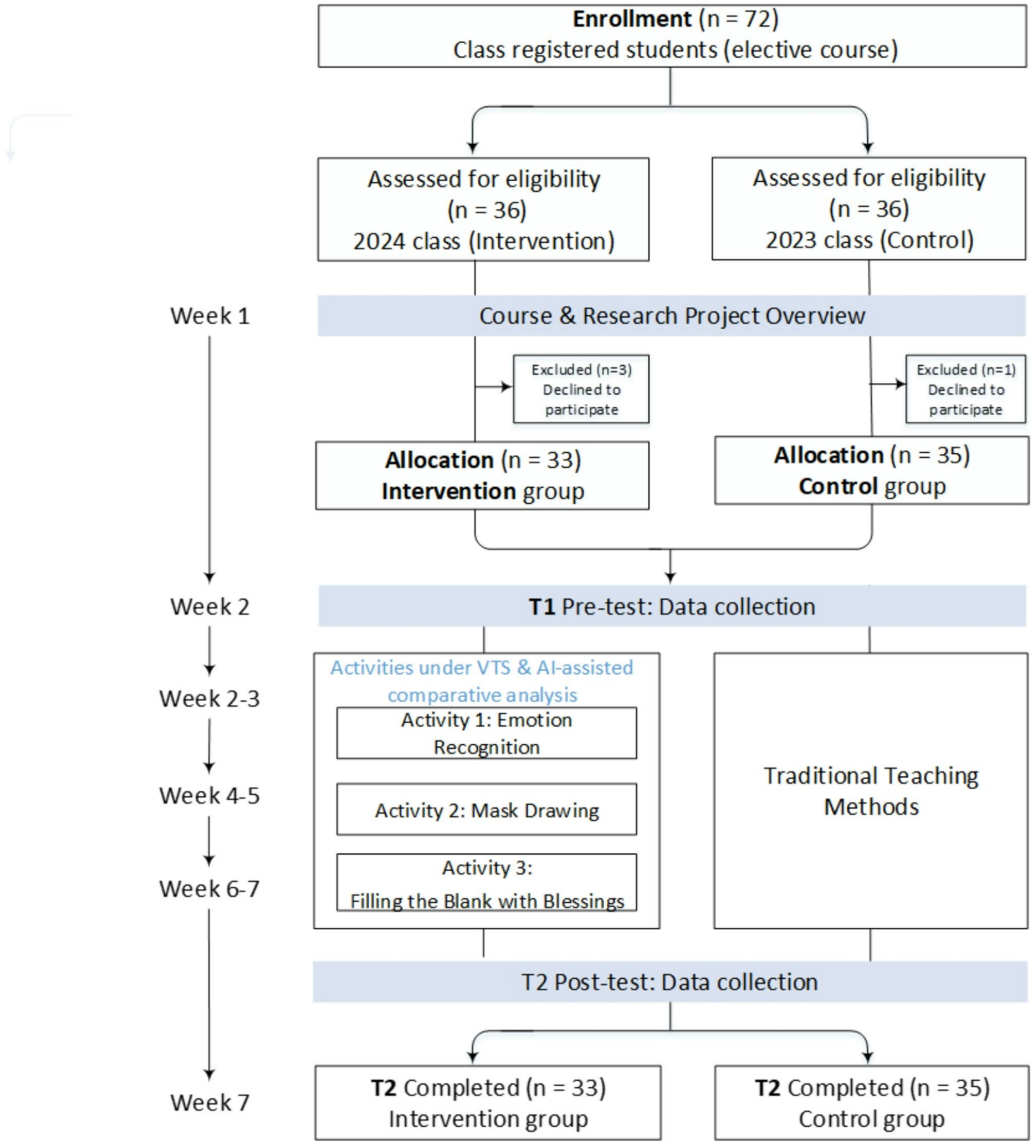
Research process flowchart showing recruitment, group allocation, intervention implementation, and data collection schedule

This is a quasi-experimental study involving medical students enrolled in an elective course titled “Interpersonal Communication and Patient-Physician Relationships” in 2023 and 2024 (Fig. 1.). Students studying this elective course in 2023 were assigned to a control group (*n* = 35); those studying in 2024 were assigned to an intervention group (*n* = 36). The control group received traditional teaching methods, while the intervention group experienced VTS with AI-assisted comparative analysis. Inclusion criteria required that participants agreed to participate in the research and attended all intervention activities. Students who did not complete all activities were excluded from the study. Students who declined participation (3/36; 8.3% of the 2024 experimental group) were excluded from analysis. Participants provided written informed consent and permission to publish their drawings and written responses. The study received ethics approval from the National Cheng Kung University Human Research Ethics Committee (112–739).

### Measures

Self-emotion appraisal (SEA) and others’ emotion appraisal (OEA). Adapted from Wong and Law, SEA and OEA each consisted of four items rated using a seven-point Likert scale to assess individuals’ ability to understand their own emotions and observe others’ emotions [28]. Sample items are “I have a good sense of why I have certain feelings most of the time” (SEA) and “I am a good observer of others’ emotions” (OEA). All items were scored with higher scores indicating better appraisal ability, with both scales showing excellent internal consistency across pre and post assessments (SEA: α = 0.87–0.88; OEA: α = 0.91–0.94). The item scores were then averaged to present overall SEA and OEA scores.

Empathy. Adapted from Toronto Empathy Questionnaire, consisted of fifteen items rated on a five-point scale (0 = never to 4 = always) to assess individuals’ empathetic responses to others’ emotional states [29]. Sample items include “When someone else is feeling excited, I tend to get excited too” and “I can tell when others are sad even when they do not say anything.” All items were scored with higher scores indicating greater empathy (with reverse coding for negatively worded items), and the scale demonstrated excellent internal consistency across pre and post assessments (α = 0.82–0.86). The item scores were then averaged to present an overall empathy score.

Student well-being. Seven items with dichotomous responses (yes/no) were used to assess students’ physical and emotional well-being. A sample item is “Do you feel burned out from schoolwork?” All items were scored with higher scores indicating poorer well-being, and the scale demonstrated good internal consistency (α = 0.67 pre, 0.77 for post scores) were then summed to present an overall well-being score. These questions were adapted from the medical student well-being index [30].

### Intervention procedures

The intervention consisted of six weeks of art-based activities (two hours per week). Using VTS as an interpretive framework, participants uploaded artwork to AI platforms, corrected AI interpretations, and compared these with peer feedback and original intent. All activities used AI-assisted analysis within a VTS framework, where students uploaded artwork to platforms like ChatGPT [11] or Claude [31], compared AI interpretations and peer feedback with their original intent, and validated or corrected both interpretations, along with reflection on the comparative analyzing process. Full pedagogical rationales, complete step-by-step protocols, and photographic examples are provided in Supplementary material 1.

**Fig. 2:**
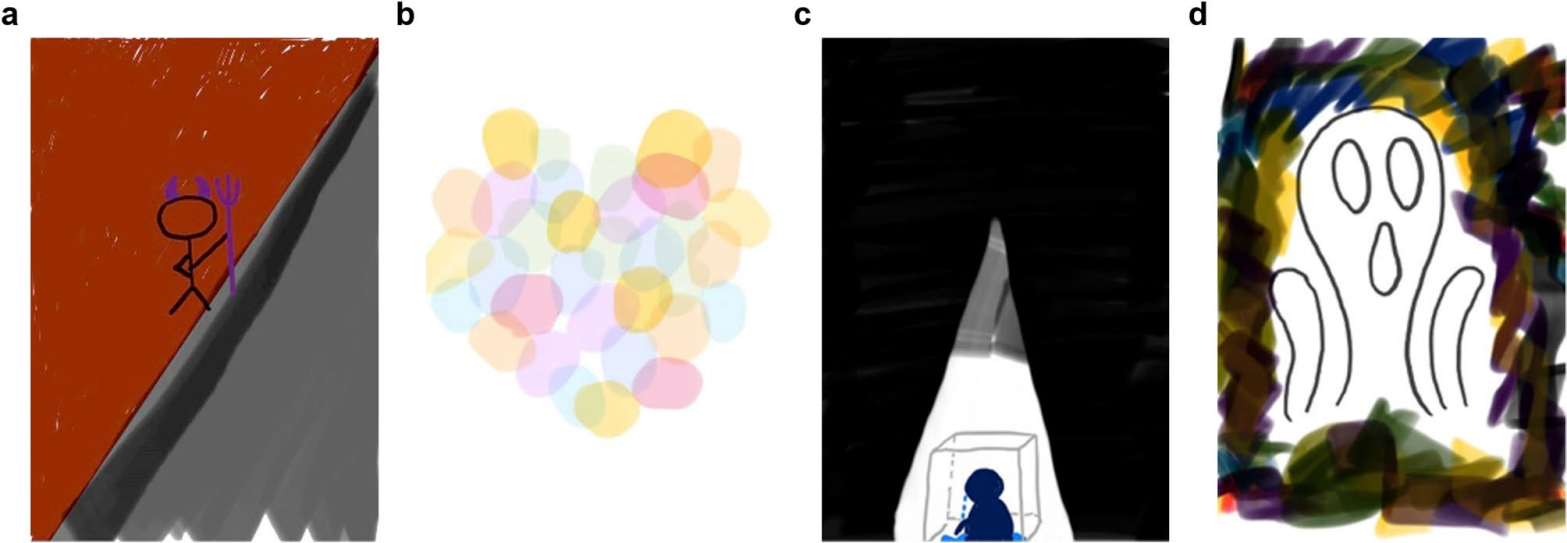
Participants’ drawings of four emotions from intervention activity one. Notes: Participants’ interpretations of their emotional drawings are as follows: **a**: Red symbolizes anger - when thoughts become chaotic and dark with negative feelings, that's why I represent it with a little demon. **b**: Happiness is composed of beautiful memories, and while these images may gradually fade, they leave colorful lasting impressions. **c**: Sadness emerges from feeling misunderstood, like being trapped alone in a tiny space within complete darkness, drawing circles in solitude. **d**: The figure references "The Scream," depicting fear through facial expression, with surrounding dim colors representing unknown fears

In Activity 1 (Emotion recognition), participants received a blank JPG file sent to their smartphones and used any familiar digital editing tool to create visual representations of four fundamental emotions (anger, happiness, sadness, fear) within 30 min, emphasizing emotional expression rather than artistic technique. Following the creation phase, participants engaged in structured group discussions where peers guessed the emotion depicted, followed by the creator’s explanation of their visual choices. The activity concluded with a reflective writing assignment in which students analyzed how visual elements could effectively communicate different emotional states. Selected art drawings are shown in Fig. 2a to d. Detailed instructions for Activity 1 have been previously published in [32].

**Fig. 3:**
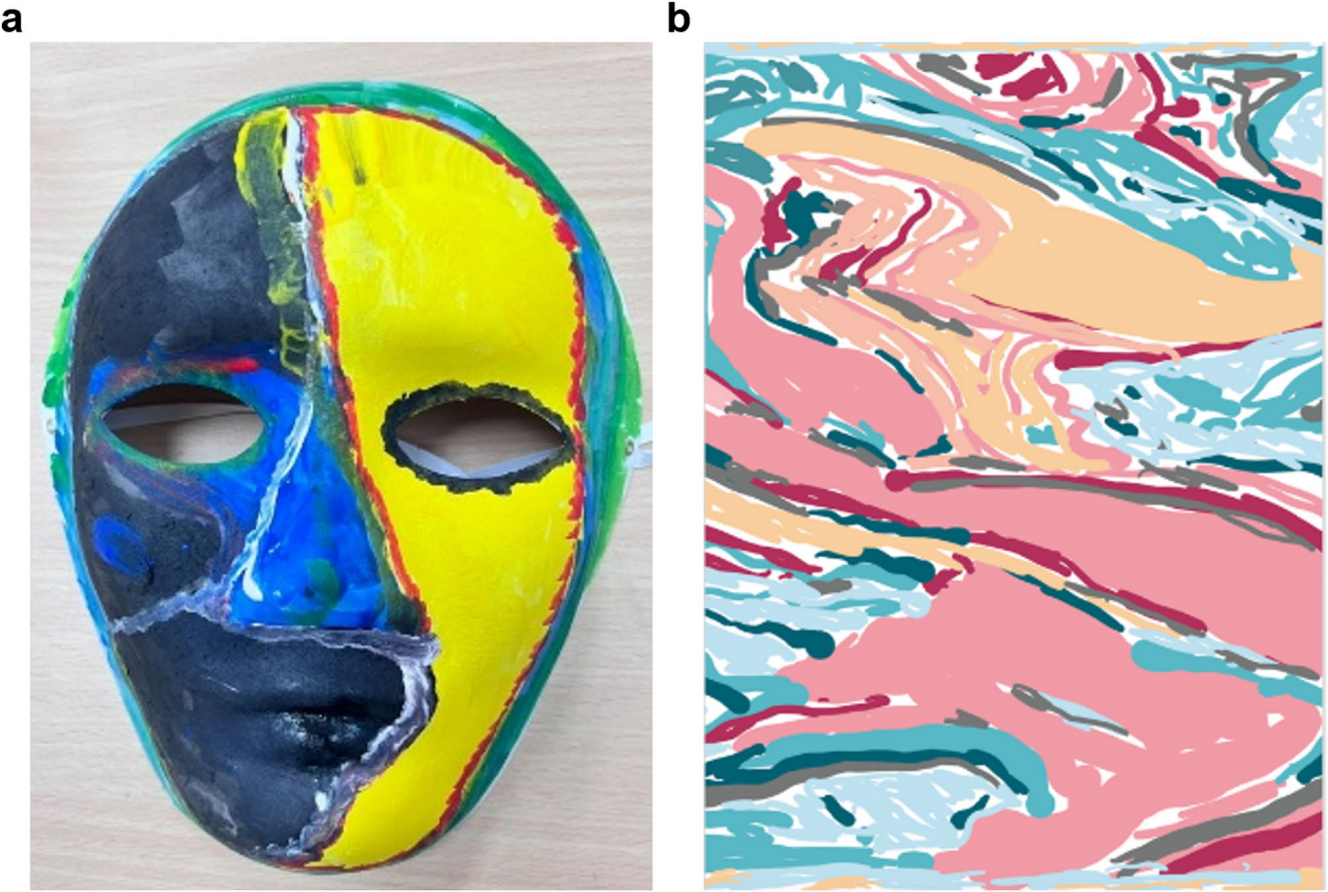
Selected participant’s drawing representing from activity two. **a**: Persona. **b**: Inner self

Activity 2 (Emotional mask drawing), inspired by Jung’s persona and shadow concepts [33], began with a short introduction to Jung’s “persona” and “shadow” and Freud’s “Ego, Id, and Superego” to provide a theoretical foundation. Participants created physical masks with acrylic paint to symbolize their public personas, followed by abstract digital drawings using geometric shapes, dots, or lines to represent their private inner selves. The classroom transformed into a museum space where artworks were displayed and uploaded to a private online platform for peer review. Each student provided written feedback on at least six peers’ works. For homework, students uploaded their artworks to an AI platform to answer VTS questions, corrected AI interpretations, and wrote reflections comparing peer feedback, AI responses, and their own intentions. Selected art drawings are shown in Fig. 3a and b. These illustrations show how mask drawing addressed key outcomes: contrasting public personas with private selves fostered self-emotion appraisal; sharing and interpreting peers’ masks strengthened others’ emotion appraisal; and exploring persona–shadow contrasts promoted empathy through recognition of shared experiences. Comparing AI and peer interpretations was used to assess the added value of human feedback in AI-assisted learning, with deeper insights at higher VTS levels reported in the Results.

**Fig. 4:**
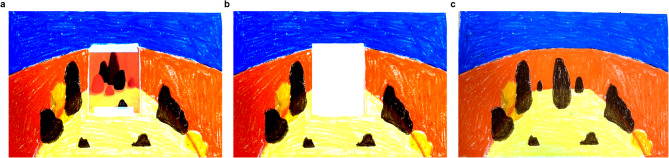
Selected participant's drawing representing from activity three. **a**: Initial mood selection and personal expression. **b**: Creative space and transformation. **c**: Collaborative artistic exchange

In Activity 3 (Filling the blank with blessings), participants chose an art card representing their mood from the past week and created drawings inspired by it. The card was then removed, leaving an empty space on the canvas for a partner to add a “canvas blessing” – a positive thought or wish expressed visually. This activity was designed to develop empathy and emotional awareness through paired discussions where participants shared their artwork, explained the meaning of the blessings, and reflected on the emotional experience of giving and receiving them. Selected drawings are shown in Fig. 4a to c. These illustrations show how the blessing activity fostered key outcomes. Mood reflection and visualization supported self-emotion appraisal, while interpreting and responding to partners’ additions enhanced others’ emotion appraisal. The exchange of blessings promoted empathy through considering peers’ experiences and contributed to student well-being by reinforcing positive, supportive interaction.

Across all activities, the comparative analysis of AI, peer, and self-interpretations was designed to stimulate metacognitive engagement by prompting students to reflect on the recognition and perception of both their own and others’ emotions. This process encouraged learners to monitor their interpretative strategies, assess accuracy, and adjust their emotional reasoning in response to feedback.

### Data collection

Data were collected from three primary sources: (1) pre-post assessments measuring changes in EI, empathy, and well-being; (2) participants’ artwork, art making intention, and written reflections; and (3) interpretative data, including AI platform analyses of participants’ artwork and peer feedback, where each participant selected six images that elicited the strongest emotional response and provided written feedback on these selections. The limitation to six images for peer feedback was implemented to prevent feedback fatigue, maintain response quality, and ensure consistent engagement throughout the process. This number balanced cognitive load constraints while enabling thoughtful feedback and encouraging critical evaluation skills as participants selected images that evoked the strongest emotional responses, enhancing the reflective component of the intervention.

#### Data analysis

In order to eliminate the artifact effects from potential confounders (i.e., baseline scores, age, and gender), repeated-measures analysis of covariance (ANCOVA) was used. Specifically, the repeated-measures ANCOVA constructed in the present study tested the group × time interaction, while adjusting for baseline scores, age, and gender to account for initial differences and demographic effects. This allowed us to determine whether the two groups changed differently over time after considering their starting scores and demographics [34]. Compared with repeated-measures ANOVA or paired t-tests, ANCOVA provides a more accurate estimate of the intervention’s effect size after accounting for covariates. In the repeated-measures ANCOVA [34], group (intervention vs. control) was the between-subjects factor and time (pre vs. post) was the within-subjects factor. The dependent variable was the post-intervention score for each emotional ability measure, with the corresponding pre-intervention score, age, and gender included as covariates. The analysis was performed using the General Linear Model procedure in SPSS 24.0 (IBM Corp., Armonk, NY) [35], which produced adjusted means and tested the interaction between group and time after controlling for covariates. For qualitative analysis, we focused on students’ reflective writings from Activity 2, as it provided the most suitable context for comparing AI and peer interpretations. Activity 2 enabled deeper analysis compared to Activity 1, moving beyond basic emotional perception to more complex interpretative processes. Furthermore, unlike Activity 3, where students interpreted others’ exchanged artworks, Activity 2 allowed students to analyze their own creations, giving them authentic knowledge of their creative intentions.

## Results

**Fig. 5:**
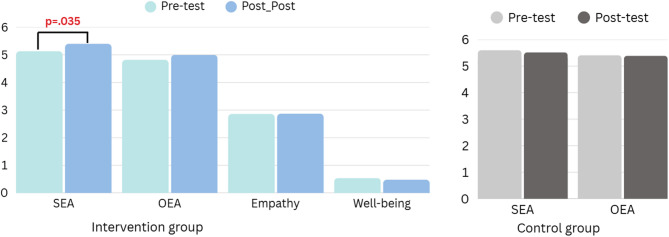
Self-Reported Survey Score Changes: Intervention vs. Control Groups (T1 VS. T2)

Table 1 shows participant demographics: ages 18–22 (mean = 19.85, SD = 1.15), with more males (63.6%) than females (36.4%). Table 2 compares EI outcomes between groups. SEA improved significantly in the intervention group (pre 5.13 VS post 5.40, *p* = 0.035) but not in controls (*p* = 0.501; Fig. 5). The group × time interaction for SEA was significant (*p* = 0.027). OEA showed no changes, and neither empathy nor well-being improved within the intervention group.

**Table 1:** Demographic characteristics of study participants: intervention and control groups

	Intervention group (*n* = 33)		Control group (*n* = 35)		
	Mean or n	SD or %	Mean or n	SD or %	p
Age	19.85	1.15	19.34	0.96	0.407
Sex					0.492
Female	12	36.40	10	28.57	
Male	21	63.60	25	71.43	

Age ranges between 18 and 22 years

**Table 2:** Results of pre-post comparison tests and repeated measures ANCOVA for experimental and control groups with age and sex controlled

	Intervention group					Control group							
	Pre-test		Post-test			Pre-test		Post-test			Group *Time Interaction		
	Mean	(SD)	Mean	(SD)	*p* ^a^	Mean	(SD)	Mean	(SD)	*p* ^a^	*F*	*p*^b^	partial η²
Self-emotion appraisal	5.13	(1.14)	5.4	(0.91)	0.035	5.60	(1.10)	5.52	(1.19)	0.501	5.14	0.027	0.074
Others’ emotion appraisal	4.82	(1.16)	5.0	(1.09)	0.095	5.41	(1.24)	5.39	(1.16)	0.813	1.23	0.271	0.019
Empathy	2.86	(0.44)	2.87	(0.49)	0.729	--	--	--	--	--	--	--	--
Student well-being	0.54	(0.25)	0.48	(0.28)	0.130	--	--	--	--	--	--	--	--

*SEA *Self-emotion appraisal, *OEA *Others’ emotion appraisal

^a^ p-values for within-group pre-post comparisons. SEA showed significant improvement in the experimental group (*p* = 0.035) but not in control group (*p* = 0.501). OEA (*p* = 0.095), Empathy (*p* = 0.729), and Student Well-being (*p* = 0.130) showed no significant changes in the experimental group

^b^ Interaction between time (pre-test, post-test) and groups (experimental, control) was significant for SEA (F[1,64] = 5.14; *p* = 0.027) with a medium effect size (partial η²=0.074). The experimental group showed improvement from pre-test to post-test while the control group showed a slight decrease. Interaction between time (pre-test, post-test) and groups (experimental, control) was not significant for OEA (F[1,64] = 1.23; *p* = 0.271) with a small effect size (partial η²=0.019)

Table 3 presents a comparative analysis of how AI and human peers interpret artwork across the three levels of VTS. At the observation level, AI demonstrated precise attention to visual details and systematic analysis, while peers focused more on holistic impressions and emotionally salient features. In interpretation, AI often relied on universal symbolic meanings (e.g., associating blue with sadness), whereas peers drew upon shared cultural contexts and personal experiences, particularly when interpreting student-specific symbols such as clowns representing academic stress. At the level of deep insights, AI tended to provide neutral, data-driven psychological analyses and identify potential unconscious patterns, while peers excelled in detecting subtle emotional nuances and contextualizing interpretations through authentic social interactions, as reflected in medical student testimonials.

**Table 3:** Comparative analysis: student reflections on AI vs. Peer Art interpretation across three VTS levels (Mask drawing Activity)

		AI (Machine-Based Interpretation)	Human (Peer-Based Interpretation)
1. Observation	Concepts	• Detail Precision • Systematic Analysis • Objective Stance	• Holistic Perception • Selective Attention • Experiential Response
	Student Quotes	“AI observed many minor details (like white paint on the chin, which wasn’t intentional)” (ID: 10) “GPT’s analysis was quite accurate in objective aspects, precisely describing appearances based on the image” (ID: 23)	“Peers paid more attention to the overall feeling, they noticed more of the red parts” (ID: 10) “Peers’ feedback on FB, having more information about me, though less precise than GPT in objective description, excelled in expressing thoughts and emotions” (ID: 23)
2. Interpretation	Concepts	• Universal Symbolism • Element-focused Analysis • Theoretical Framework	• Emotional Connection • Cultural Context • Authentic Resonance
	Student Quotes	“When Chat-GPT interprets feature meanings, it often uses collective understanding (like blue for sadness, red for passion, tears for sorrow, stars for hope)” (ID: 25)	“Both peers and I understand the helplessness and predicament represented by the clown, and the fatigue from exams and note-taking” (ID: 22) “Classmates are companions in my life, their observations come from real interactions” (ID: 26)
3. Deep Insight	Concepts	• Pattern Recognition • Neutral Perspective • Data-driven Insights	• Emotional Sensitivity • Shadow Detection • Contextual Understanding
	Student Quotes	“Chat GPT thought very positively when making associations… ChatGPT itself was initially set to be more positive and healthier” (ID: 5)	“Human expression was partly related to direct feelings and intuition, which could not be understood through pure rational thinking” (ID: 18)

These thematic differences underscore how AI and peer contributions complement each other in meaningful ways. AI’s objective detail recognition and consistent application of symbolic frameworks can help students strengthen visual literacy and develop systematic analytical skills. Peer feedback, with its emotional resonance, cultural grounding, and supportive tone, fosters empathy, perspective-taking, and contextual reasoning—skills essential for patient-centered care. Engaging with both feedback types encourages students to integrate analytical precision with emotional understanding, thereby reinforcing metacognitive engagement in reflecting on their own and others’ emotions.

## Discussion

### Fostering emotional competency: a multi-modal approach

SEA improved significantly (*p* = 0.035), suggesting that art-based activities with VTS help bridge the expression–recognition gap. Student drawings promoted emotional engagement by enabling recognition and expression of feelings, consistent with prior findings [20, 21]. Sharing further enhanced empathy through listening and exchange, aligning with Levenson and Ruef’s stages of recognizing, experiencing, and reacting to others’ emotions [36]. Data were structured across three VTS levels: observation (neutral description, consistent with Mayer’s EI and empathy recognition [36, 37]; interpretation (evidence-based analysis supporting higher-order thinking); and deep insight (critical thinking relevant to clinical reasoning [23, 25].

### Digital native learning preferences and technology integration

Digital native medical students engaged effectively with both technological and traditional methods. Their ability to shift between AI analysis and peer feedback supports Prensky’s view of digital native learning preferences [9]. By comparing AI, peer, and self-responses, students expressed authentic emotions and clarified their drawing intentions. The design provided personalized, controllable elements with immediate feedback, aligning with Khan’s description of a paradigm shift that balances technology with essential human interaction [10].Reflective use of visual tools also enhanced higher-order thinking by engaging multiple cognitive processes, as Raiyn (2016) notes, with potential benefits for clinical reasoning [38].

The intervention’s structure may have enhanced metacognitive engagement by requiring students to actively compare their interpretations with those of AI and peers. This reflective process aligns with metacognitive models in art and design education, where deliberate monitoring of interpretative reasoning can deepen emotional understanding [26]. Although only self-emotion appraisal showed significant quantitative improvement, the qualitative data suggest that metacognitive reflection was occurring and could, over a longer intervention, also strengthen other-emotion appraisal.

### Integrating technology while preserving authentic human elements

Our intervention engaged students directly with AI analysis, allowing them to discover both its strengths in systematic analysis and limitations in emotional interpretation, which aligns with our investigation of digital natives’ emotional competency through multiple interpretive lenses. This hands-on approach revealed AI’s constraints in processing affective meaning, supporting prior research observations about AI’s limitations in emotional learning contexts [12]. Student testimonials highlighted the contrast between AI’s standardized interpretations and peers’ ability to detect subtle emotional nuances, particularly in recognizing profession-specific symbols like “clowns representing academic stress.” Peers acted as life companions, interpreting through authentic interactions and emotional resonance, supporting the need to preserve human elements in technological integration. This deep engagement approach enables students to think critically about integrating technology in future clinical settings where both authentic human care and technological efficiency are essential.

The differentiated feedback patterns observed between AI and peers carry important educational implications. AI’s systematic, detail-oriented approach supports the development of structured visual analysis, while peer feedback’s emotionally attuned and context-rich interpretations cultivate interpersonal sensitivity and cultural competence. Leveraging both sources of feedback within VTS activities allows learners to balance cognitive precision with emotional depth, a combination that is critical for effective communication and empathetic practice in clinical settings.

### Limitations and directions for future research

#### Sample size and recruitment

 With only 68 medical students from a single college, generalizability is limited. However, the quasi-experimental design, including experimental and control groups with pre–post assessments, strengthened internal validity despite the modest sample. Class size was intentionally kept small to preserve instructional quality in this qualitative, creation-centered curriculum, further constraining recruitment. A gender imbalance (36.4% female), reflecting enrollment patterns in Taiwan (30.1–45.2%; [39]) and the U.S. physician workforce (37.1%; [40]), may nonetheless shape outcomes tied to gendered perspectives [41]. Recruiting from one institution ensured demographic homogeneity and reduced curricular variability, thereby enhancing internal validity, but restricted external applicability. Future research should expand to larger, multi-institutional, and international cohorts to capture diverse cultural and educational contexts.

#### Intervention duration

 The six-week intervention may have been too short to produce lasting changes. Evidence suggests longer programs are more effective; for example, a nine-month art intervention achieved greater burnout reduction compared with controls (Volpe) [42]. A systematic review also found that among ten studies with VTS interventions, only one reported increased empathy, though with major limitations such as a single 90-minute session, subjective measures, and no control group [43, 44]. Longer interventions allow students to develop sustained habits and shift from requirement-driven to intrinsically motivated participation. In our study, however, the use of a control group and a rigorous standardized protocol helped strengthen internal validity despite the shorter duration.

#### Non-significant outcomes

 Empathy and well-being showed no statistically significant changes, likely due to the short duration, the multidimensional nature of these constructs, and possible indirect or delayed effects. The moderate sample size may also have limited statistical power. Future research should extend the intervention, include follow-up evaluations, and use broader, performance-based measures (e.g., behavioral empathy observations, qualitative patient feedback) to capture more nuanced outcomes.

#### Self-reporting bias

 While the study relied on self-reported questionnaires for measuring EI, empathy, and well-being, potentially introducing social desirability bias, several design elements strengthened its validity. The pre-post design with a control group helped isolate the intervention’s effects, and the mixed-methods approach combining quantitative and qualitative data provided triangulation to mitigate potential biases.

### Study implications

The implications of the study are, first, that combining VTS with AI-assisted art analysis effectively develops medical students’ emotional competency and visual literacy skills. Second, students can successfully integrate both AI tools and peer feedback in their learning, suggesting a viable hybrid approach. Third, while AI provides systematic analytical support, human interpretation remains essential for capturing emotional nuances and medical context, emphasizing the importance of preserving human elements in medical education, particularly in emotional learning and professional development.

## Conclusions

This study illustrates how integrating AI technology with traditional learning methods can support the development of emotional competencies in medical education. The blended use of VTS and AI-assisted art analysis improved students’ emotional appraisal and highlighted the complementarity of technological tools and human interaction. This model offers a promising framework for aligning technological innovation with the enduring value of human engagement in training healthcare professionals. Incorporating metacognitive reflection into AI-assisted VTS may further strengthen students’ capacity to monitor and refine emotional reasoning.

## Supplementary Information

Supplementary Material 1.

## Data Availability

This data has been coded and permanently de-identified. The datasets generated and/or analyzed during the current study are available in the OSF repository at. 10.17605/OSF.IO/GB8JD.
