# Supplementary material for: AI-human collaborative approaches in emotional training: applying visual thinking strategies as an analytical framework for medical students’ drawings from art-based activities
Source: BMC Med Educ. 2025 Dec 3;26:26. doi: 10.1186/s12909-025-08165-9 (PMC12781445; doi:10.1186/s12909-025-08165-9)
Supplement: Supplementary file 1 — Supplementary Material 1. [file 12909_2025_8165_MOESM1_ESM.docx]

Appendix

Appendix A: Detailed Activity Protocols

**Activity 1: Emotion Recognition**

Developmental Focus:

The emotion recognition activity aimed to develop multiple facets of emotional intelligence and empathy. Through the process of creating emotional representations, participants enhanced their self-emotion appraisal abilities as they connected their understanding of emotions with visual expression. During group discussions, participants developed other-emotion appraisal skills while interpreting peers' drawings, fostering cognitive empathy through the exchange of perspectives on how different emotions could be visually represented.

Implementation Protocol:

1. Materials and Setup

Participants received a blank JPG file sent directly to their smartphones, establishing a standardized starting point for the digital art activity. The exercise was designed to be completed within a focused 30-minute timeframe, ensuring spontaneous and intuitive responses from the participants.

2. Creative Expression Guidelines

Using any familiar digital editing tool of their choice, participants were tasked with creating visual representations of four fundamental emotions: anger, happiness, sadness, and fear. The emphasis was placed on emotional expression rather than artistic technique, encouraging participants to focus on conveying feelings rather than achieving technical perfection.

3. Group Sharing Process

Following the creation phase, the classroom transformed into a collaborative learning space where participants engaged in a structured group discussion. Group members first attempted to guess which emotion each drawing represented. Then, the presenting participants explained their creative choices and reasoning behind their emotional representations. This interactive sharing session provided an opportunity for participants to articulate their thought process while learning from both their peers' interpretations and different artistic approaches.

4. Reflective Learning

The activity culminated in a reflective writing assignment to be completed as homework. Participants documented their insights about emotional expression and recognition gained through the exercise, analyzing how visual elements could effectively communicate different emotional states. This written reflection helped solidify their understanding of the relationship between visual art and emotional communication.

**Activity 2: Mask Drawing**

Developmental Focus:

The mask drawing activity cultivates deep self-awareness and interpersonal understanding through artistic expression. As participants create their masks, they develop self-emotion appraisal skills by examining the distinction between their public and private selves. The process of sharing and providing feedback on others' works enhances other-emotional appraisal abilities, while the comparison between persona and shadow representations develops empathy through recognition of shared human experiences.

Implementation Protocol:

1. Theoretical Framework and Psychological Concepts

The activity began with an introduction to Jung's "persona" and "shadow" and Freud's "Ego, Id, and Superego" concepts, providing the theoretical foundation for participants' creative expressions.

2. Two-Part Creative Process

Participants engaged in a two-part creative process: creating masks with acrylic paint to symbolize their public personas, followed by producing digital drawings of their inner selves using abstract geometric shapes, dots, or lines with optional color incorporation.

3. Museum Experience and Peer Feedback

The classroom transformed into an art museum displaying the masks, with artworks uploaded to a private Facebook group. Participants toured the exhibition as museum visitors and provided written feedback on at least six peers' artworks through the platform.

4. Reflective Learning

For homework, participants uploaded their artworks to an AI platform to engage with Visual Thinking Strategies questions, correcting AI interpretations where necessary. They then wrote reflections comparing three perspectives: peer feedback, AI responses, and their own authentic interpretations as creators.

**Activity 3: Filling the Blank with Blessings**

Developmental Focus:

This activity develops emotional awareness and empathetic connection through the exchange of artistic blessings. Participants enhance their self-emotional awareness through initial mood reflection and semester experience visualization. The blessing exchange process cultivates both affective and cognitive empathy as students consider their partners' experiences and emotions while creating supportive artistic messages. This mutual exchange of positive intentions helps develop skills in other emotional appraisal and emotional use in social contexts.

Implementation Protocol:

1. Initial Mood Selection and Personal Expression

The protocol began with participants selecting an art card that resonated with their experiences from the past week. Using this selected card as inspiration, they created drawings that visually expressed their emotional state and mood.

2. Creative Space and Transformation

The art cards were then removed, intentionally leaving an empty space on each participant's canvas. This vacant area served as a designated space for the next phase of artistic interaction.

3. Collaborative Artistic Exchange

Participants were paired together for a reciprocal creative exercise. Each participant created a 'canvas blessing' in their partner's empty space, focusing on positive thoughts or wishes for their peer.

4. Reflective Learning

The activity concluded with paired discussions where participants shared their artwork, explained the inspiration behind their blessings, and explored the emotional resonance of both giving and receiving artistic support.

|  |  |  |
| --- | --- | --- |
| Figure1a. Initial Mood Selection and Personal Expression | Figure 1b. Creative Space and Transformation | Figure 1c. Collaborative Artistic Exchange |

Figure 1 Selected participant's drawing representing from activity three (ID 17)
